# Do Housekeeping Genes Exist?

**DOI:** 10.1371/journal.pone.0123691

**Published:** 2015-05-13

**Authors:** Yijuan Zhang, Ding Li, Bingyun Sun

**Affiliations:** Department of Chemistry and Department of Molecular Biology and Biochemistry, Simon Fraser University, Burnaby, British Columbia, Canada; Georgia Institute of Technology, UNITED STATES

## Abstract

The searching of human housekeeping (HK) genes has been a long quest since the emergence of transcriptomics, and is instrumental for us to understand the structure of genome and the fundamentals of biological processes. The resolved genes are frequently used in evolution studies and as normalization standards in quantitative gene-expression analysis. Within the past 20 years, more than a dozen HK-gene studies have been conducted, yet none of them sampled human tissues completely. We believe an integration of these results will help remove false positive genes owing to the inadequate sampling. Surprisingly, we only find one common gene across 15 examined HK-gene datasets comprising 187 different tissue and cell types. Our subsequent analyses suggest that it might not be appropriate to rigidly define HK genes as expressed in all tissue types that have diverse developmental, physiological, and pathological states. It might be beneficial to use more robustly identified HK functions for filtering criteria, in which the representing genes can be a subset of genome. These genes are not necessarily the same, and perhaps need not to be the same, everywhere in our body.

## Introduction

The study of gene function and organization has been a fundamental goal in molecular and cellular biology. The outcome not only benefits our understanding on health and disease, but also provides critical information for bioengineering of novel systems that can better serve our needs. The emergence of high-throughput transcriptomic techniques enables direct analysis and comparison of gene expression across different biological samples. Among these comparative studies, the interest of seeking housekeeping (HK) genes in tissues of multicellular organisms has been widely focused for the maintenance of basal and essential cellular functions [1, 2].

The definition of HK genes implies that except for species, little biological impact should be exerted to the discovery of these genes, because these genes are expressed irrelevant to the tissue type, developmental status, cell-cycle state, or external environment [3]. Such robust expression will likely warrant an easy detection of HK genes regardless of the detection methods, such as microarray and sequencing techniques [4]. Currently, more than a dozen studies have been conducted on human HK genes, but the sampling depth varies [2, 3, 5–17]. Because all of the studies include certain false positive HK genes due to limited sampling, we hypothesize that an integration of the results will increase the sampling coverage of human body and effectively remove the false positives.

Based on estimation, human has around 200 tissue and cell types[6], yet in all the HK-gene studies carried out so far, none of them covered more than half of these types to our knowledge. The reverse correlation of HK-gene number and the tissue coverage (i.e. Expression Breadth, EB) has been demonstrated in numerous studies [18–20]. As a result, incomplete tissue coverage will introduce non-authentic HK genes, which can be eliminated by combining results derived from different studies. To test this hypothesis, we merge here 15 human HK-gene lists obtained from the public domain[2, 3, 5–15] to increase the tissue coverage (> 90% with more than 180 tissue and cell types included). The results are surprising and interesting: for a total of more than 12,500 HK genes obtained, only one gene is shared by all the studies, and 17 genes are in 14 out of 15 datasets. We ask whether the observed small number suggests that there could be no HK genes; or suggests that other factors, such as the detection methods and the filtering criteria of HK genes could prevent us from recognizing them.

To seek answers, we describe here in detail the steps we took to study these datasets, including the comparison of the used samples, the analysis technique, and the stringency of the applied filtering criteria. In addition, we examined the biological functions enriched in these lists. Based on the obtained information, we will discuss in the end the possible explanations to our observation and hope the findings could assist future studies.

## Data collection and analysis

### Data collection and processing

We obtained 15 different human HK gene lists from the public domain (Table 1). The source from which the data was downloaded is summarized in S1 Table. To compare different lists, we first unified their identifiers to Entrez Gene ID using the DAVID Bioinformatics Resources 6.7[21]. For lists already using Entrez Gene ID, we also updated their indices using DAVID to eliminate any potential inconsistency caused by the evolving database. If multiple IDs were mapped, all of them were considered to maximize the chance of finding overlapping genes. Any redundancy generated from the conversion was removed.

**Table 1:** Summary of HK gene studies*.

List	Original gene No.	Gene ID No.	Technique	Deviation ranking	HK definition	EB/REB	Year	Ref.
**Warrington**	533	754	MA	8	Ⅰ	11/100%	2000	14
**Hsiao**	451	668	MA	7	Ⅰ	19/100%	2001	15
**Eisenberg_03**	575	714	MA	5	Ⅰ	47/100%	2003	2
**Tu**	1789	1844	MA	9	Ⅰ	73/92%	2006	5
**Zhu_MA**	2448	2862	MA	11	Ⅰ	18/89%	2008	6
**Zhu_EST**	6990	7593	EST	13	Ⅰ	18/89%	2008	6
**Podder**	1350	1429	EST	2	Ⅱ	35/-	2009	7
**Dezso**	2375	2830	MA	10	Ⅰ	31/100%	2008	8
**She**	1522	1867	MA	6	Ⅱ	42/98%	2009	9
**Chang**	2064	2487	MA	12	Ⅱ	43/100%	2011	10
**Shyamsundar**	5592	4211	MA	1	Ⅰ	35/75%	2005	11
**Ramskold**	8079	8121	RNA-seq	14	Ⅰ	18/100%	2009	12
**Reverter**	4006	3208	MPSS	3	Ⅰ	32/78%	2008	13
**Eisenberg_13**	3804	3945	RNA-seq	4	Ⅱ	16/100%	2013	3
**Fagerberg**	9250	8945	RNA-seq	15	Ⅰ	27/100%	2014	17

* Redundant IDs in both original and converted lists are removed, so the listed values can be different from the original publications. MA stands for microarray. EB and REB stand for Expression Breadth and relative expression breadth, respectively.

All lists except for the one from Shyamsundar et al. were directly converted by DAVID. Shyamsundar et al. used Clone ID as identifier, which was translated based on Clone/Gene ID Converter Version 2.0 (http://idconverter.bioinfo.cnio.es/) prior to DAVID processing.

### Qualitative analysis

We first generated a union list from all datasets. We then analyzed the HK level by detection breadth (DB), i.e. the number of lists, in which a gene was included. We further performed a hierarchical analysis on all the lists using Multi Experiment Viewer (MeV) version 4.9 (http://www.tm4.org/), in which we used “1” for presence and “0” for absence of a gene.

Subsequently, we performed a leave-out analysis to examine the impact of highly deviated datasets to the results. In this study, a single dataset was first randomly removed, and the number of common genes from the rest was examined. The dataset contributed the most to the increase of common genes upon removal was ranked the highest in deviation. Then, a subsequent dataset was eliminated following the removal of the most deviated one, and the common genes resolved from the rest lists were computed again to determine the second most deviated dataset. This step was repeated until the deviation ranking of all the studies was obtained.

We also carried out a pairwise comparison to all the datasets to seek similarity distribution across studies, in which the ratio between common genes and genes in the smaller list of the two was computed as similarity ratio.

### Quantitative Analysis

To examine the contribution of expression level to the observed discrepancy, we analyzed the abundance that can be obtained in 8 studies including “She”, “Chang”, “Eisenberg_03”, “Shyamsundar”, “Zhu_MA”, “Zhu_EST”, “Fagerberg” and “Warrington”. To compare, we normalized each dataset based on the highest gene expression in that list. The mean quantity of common genes shared by different lists was calculated, and the distribution of the gene expression level was analyzed as a function of gene population, as well as a function of Detection Breadth (DB, i.e. housekeeping level).

### Miscellaneous comparison

To analyze the potential cause of the decreased overlap with the increased number of lists, we further compared the sampling depth and HK-gene filtering criteria among all the studies. For sampling, we examined qualitatively the tissue and cell types included. We also studied quantitatively the sampling coverage by Expression Breadth (EB) [18–20]. To evaluate the filtering stringency, we further defined the relative expression breadth (REB) for the cutoff percentage used in each study, i.e. percentage of EB of a particular gene to the total EB of a study. For example, if HK genes were detected in all the analyzed tissues, the REB will be “1”; if HK genes were detected in 16 out of 18 studied tissues, the REB will be 89% as in the case of “Zhu_EST” and “Zhu_MA”. EB and REB together were used to evaluate the tissue coverage and filtering stringency in each study.

### Functional Enrichment Analysis

To examine the function maintained by the discovered HK genes, we used the DAVID functional annotation tool. With default threshold (i.e. gene count = 10 and EASE = 0.05, a modified Fisher Exact P-Value), we examined the enriched Biological_Process (BP) in Gene Ontology (GO) (i.e. GO_BP) of all lists. Due to the processing limit (3000 genes) in DAVID, for large lists of “Zhu_EST”, “Ramskold”, “Eisenberg_13”, “Fagerberg” and “Reverter” that were exceeding the limit, we randomly chose 3000 genes using Microsoft excel “randbetween” function. To examine the impact of DB to GO enrichment, we separately performed enrichment on high DB (>10) and low DB (= 1) genes, and compared the difference.

## Results

### Conversion of datasets

The original data was downloaded from public domain as detailed in S1 Table. During the conversion to Entrez Gene ID, most lists were changed to certain degree; and Table 1 summarizes the number of the original and converted genes. The change was caused by the mapping to multiple IDs, and the removal of the unconverted and duplicated genes. Lists already using Entrenz Gene ID experienced the smallest change. The observed changes are common to any studies involving the conversion of gene indices [22].

### Detection Breadth (DB) analysis

**Fig 1:**
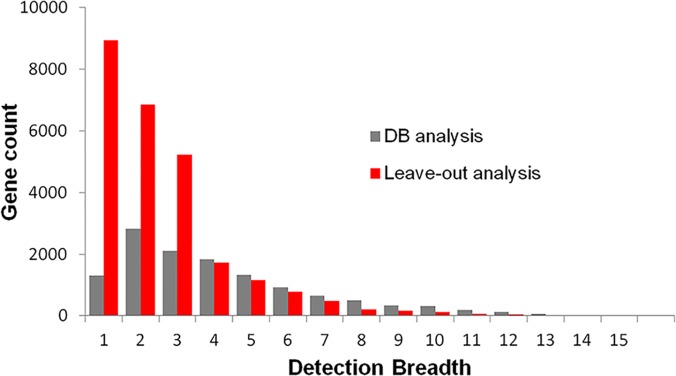
Distribution of the detection breadth (DB) among unified housekeeping (HK) genes (red bar) and the resolved common genes in leave-out analysis (grey bar).

The number of the unified HK genes from all datasets is 12,517. The distribution of these genes as a function of DB is shown in Fig 1 (red bar). In the figure, the number of genes decrease exponentially with the increase of DB value except for DB = 1 group. Only 1 gene is common to all 15 datasets. The most populated category is DB = 2, and DB = 1 represents unique genes identified only in one study. The genes with DB ≤ 3 occupy 50.0% of the union list, and only a small number of genes (3.14%) have a DB value of more than 10.

**Fig 2:**
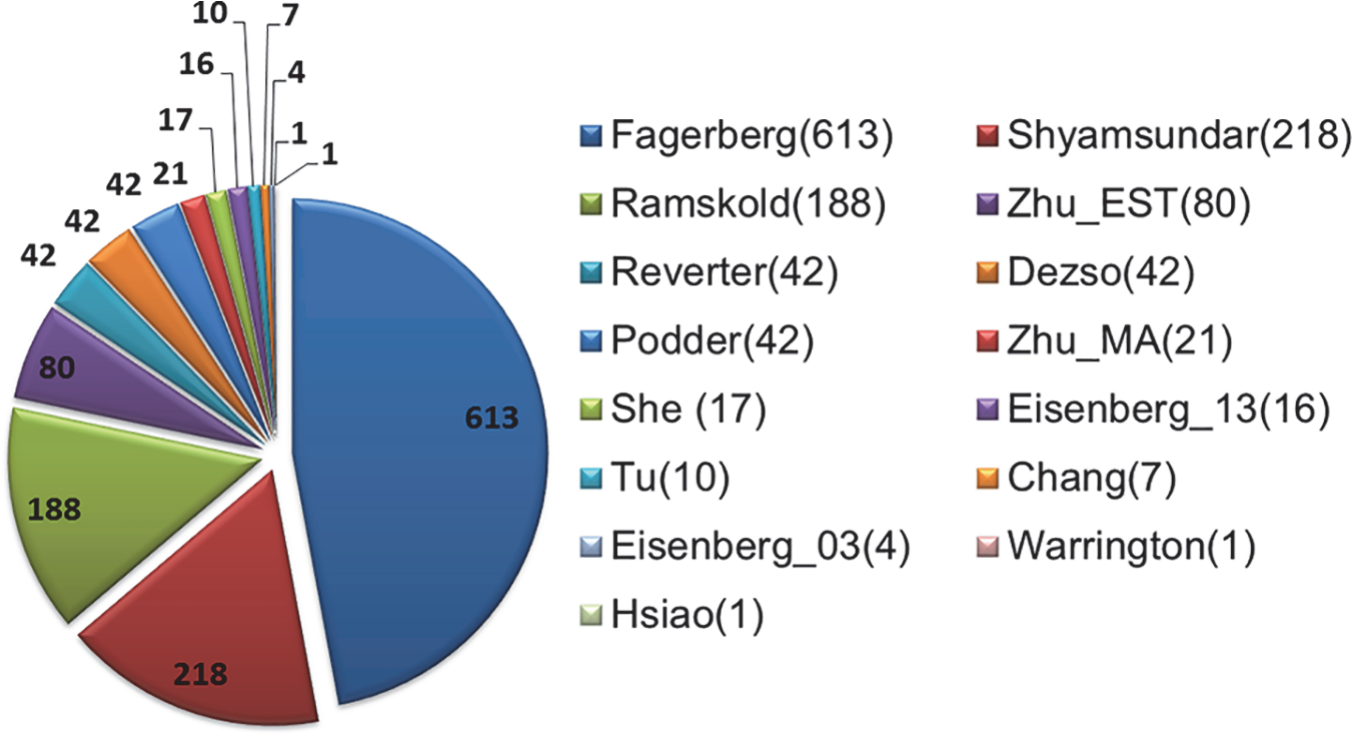
Distribution of unique genes (DB = 1) in all studies.

To examine the distribution of unique genes (DB = 1) in all studies, we plotted the number of DB = 1 genes in each study as a pie chart in Fig 2. The range of unique gene numbers is wide, from a single gene in studies of “Warrington” and “Hsiao”, to 613 genes in “Fagerberg”). This result raised our interest in analyzing the global relationship among different studies.

### Hierarchical clustering analysis

**Fig 3:**
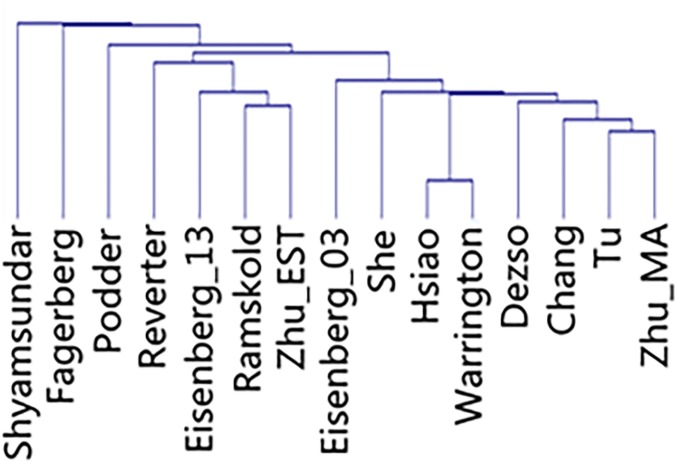
Hierarchical analysis of 15 HK-gene studies. The presence of a gene is assigned “1” and the absence of a gene is assigned “0”.

To generate the global relationship map, we conducted a non-supervised hierarchical clustering. The distance dendrogram is shown in Fig 3. Except for lists of “Shyamsundar”, “Fagerberg” and “Podder”, two main clusters with a few subclusters were observed and are listed in Table 2, according to their relative distance.

**Table 2:** Clusters by hierarchical clustering analysis.

Cluster 1	Cluster 2	
Reverter, Eisenberg_13, Ramskold, Zhu_EST	Eisenberg_03, She, Hsiao, Warrington, Dezso, Chang, Tu, Zhu_MA	
**Cluster 1.1**	**Cluster 2.1**	**Cluster 2.2**
Eisenberg_13, Ramskold, Zhu_EST	Hsiao, Warrington	Chang, Tu, Zhu_MA
**Cluster 1.1.1**		**Cluster 2.2.1**
Ramskold, Zhu_EST		Tu, Zhu_MA

### Leave-out analysis

Because the results of both unique-gene analysis and hierarchical clustering suggested the existence of highly deviated lists in all the examined studies, we wanted to know whether leaving these datasets out, would increase the consistency of the rest.

The deviation based ranking of every study obtained from leave-out analysis is listed in Table 1. The improvement on the number of common genes after the sequential removal of the most deviated studies are plotted together with the results of DB analysis as shown in Fig 1 (grey bar). In the figure, the study of leave-one-out has DB value of 14, and the study of leave-two-out has DB value of 13, and so on. The number of common genes is not growing as quickly as DB analysis at high and medium DB values (DB>7), but this trend is quickly reversed at extremely low DB values (DB < 3), when the majority of studies are left out. This observation suggests that deviation or discrepancy among HK-gene lists is ubiquitous, and only a few lists show high similarity to each other.

### Pairwise analysis

**Fig 4:**
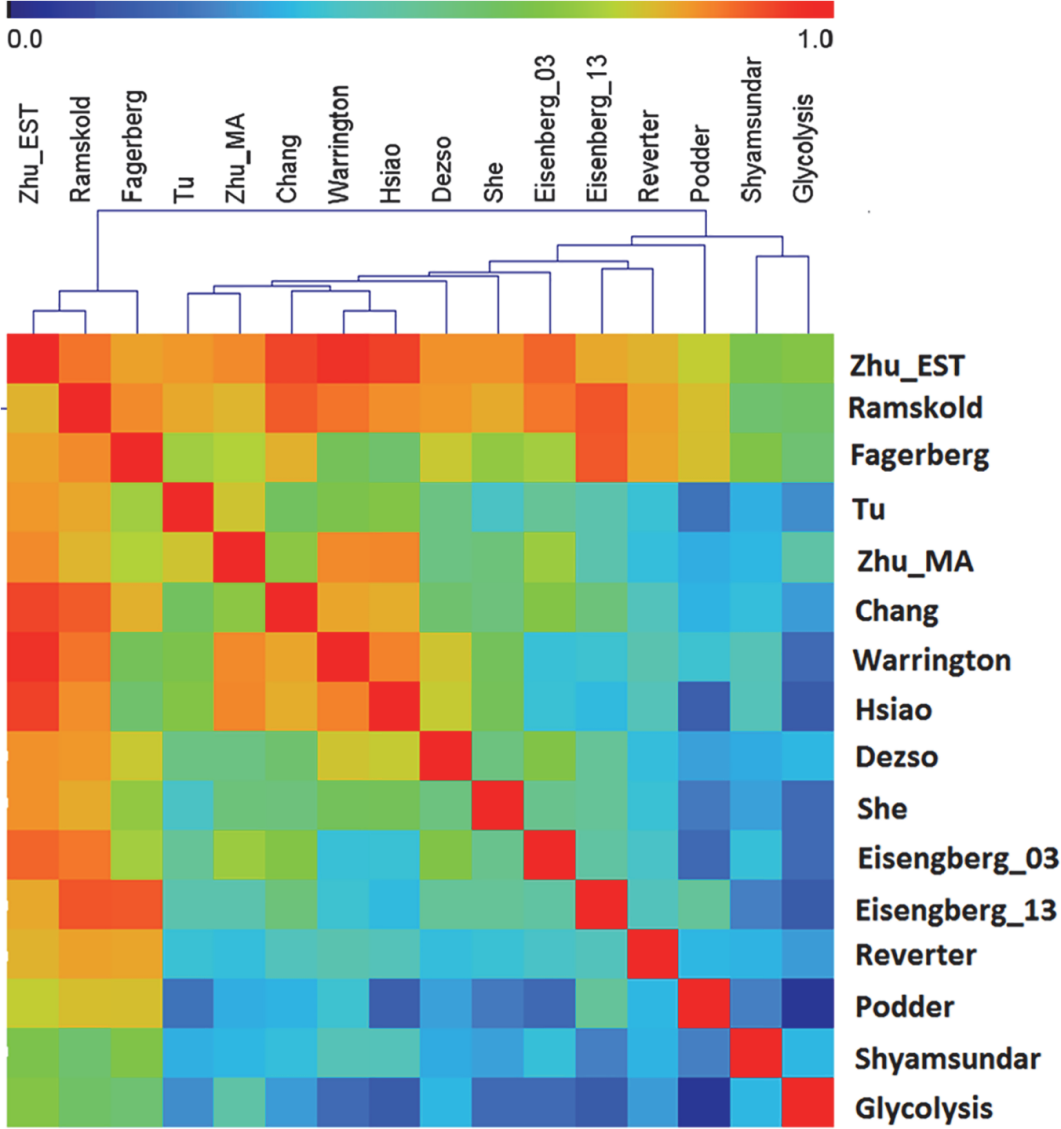
Pairwise comparison of all HK-gene lists. The color represents the similarity ratio, i.e. the ratio of the number of common genes to number of genes in the smaller list. Blue color represents a ratio of 0, and red represents 1.0.

To gain detailed information on how these results alike, we conducted a pairwise comparison to all datasets, and Fig 4 summarizes the results, in which the color encodes the similarity ratio. The distribution of this ratio is relatively small compared to the unique-gene distribution with a mean value of 0.62 ± 0.23, but local domains can be observed. For example, three relatively red bands corresponding to studies of “Ramskold”, “Fagerberg”, and “Zhu_EST” are observed. In the red bands representing high similarity ratios, the color to “Shyamsundar” is green, further suggesting its deviation from the rest.

### Abundance analysis

**Fig 5:**
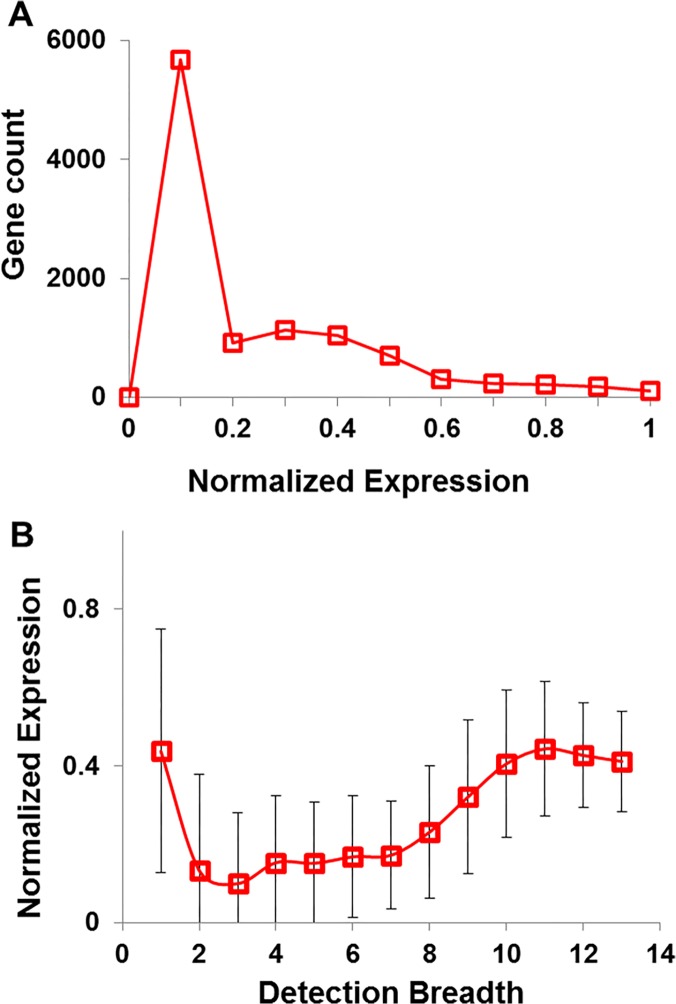
(A) Distribution of HK-gene population as a function of normalized gene-expression quantity. (B) Distribution of the detection breadth (DB) as a function the normalized gene-expression quantity. Error bar represents the standard deviation.

Fig 5A shows the distribution of gene numbers as a function of their abundance in 10,524 quantified HK genes that we were able to obtain from public domain. In the figure, gene number spikes at low quantity (normalized quantity < 0.2), suggesting the existence of large number of lowly expressed genes. Most low-quantity genes (8,932 genes) are from the “Fagerberg” list. A further analysis of the mean quantity as a function of DB is shown in Fig 5B. In the figure, a close to concave shape is observed with both low and high DB genes having relatively high abundance, suggesting the existence of a large number of unique genes is not necessarily caused by low expression and inadequate detection sensitivity. To address the other potential causes of the populated low DB genes, we examined other factors as listed below.

### Miscellaneous comparisons

We further examined the potential bias in techniques, filtering and sampling strategies. Table 1 summarizes the general information of technique and the number of tissues studied. S2 Table enlists all the tissue types, and S1 Text provides the detailed information on the used technique, software, and the filtering criteria of HK genes. The used techniques largely agreed with the clusters identified in Table 2 and Fig 3. For instance, in Table 2 and cluster 1, all the studies used sequencing based techniques, including EST, MPSS, and RNA-seq. In cluster 2, all the studies used microarray. Within cluster 1, RNA-seq and EST based studies, i.e. “Eisenberg_13”, “Zhu_EST” and “Ramskold”, are further grouped into subcluster 1.1, and among them “Zhu_EST” and “Ramskold” form the tightest subcluster 1.1.1. In Table 2 and cluster 2, two subclusters are observed that each includes studies used the same microarray platform. Specifically in cluster 2.1, both “Warrington” and “Hsiao” used the HuGeneFL GeneChip Array; similarly in cluster 2.2, all studies of “Zhu_MA”, “Tu” and “Chang” used microarray results published by Su et al. [23]. Among them, “Tu” and “Zhu_MA” are the closest and further grouped to cluster 2.2.1.

**Fig 6:**
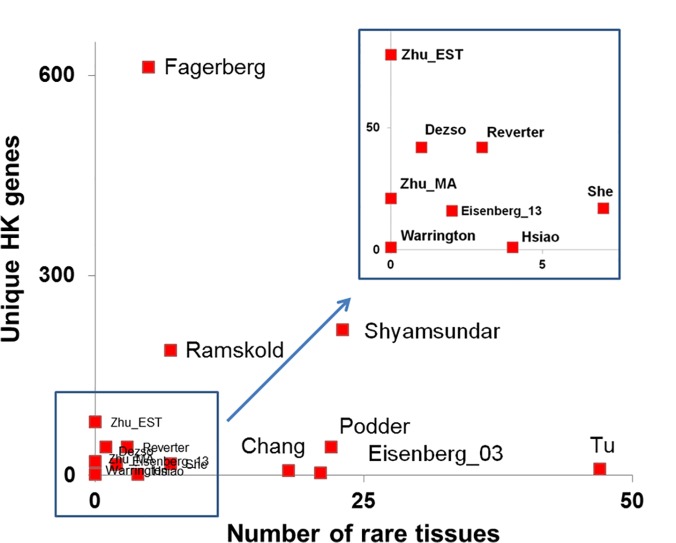
Distribution of number of unique genes (DB = 1) and the number of rare tissues, i.e. tissues used by less than 2 studies. The square region is amplified for better viewing.

We also examined the tissue types used in these studies as summarized in S2 Table. In the table, we counted the frequency of each tissue type used in all studies. In total, 187 distinct tissue and cell types were studied. These types included both adult and fetal tissues at normal or cancerous stages. Tissues and cells that were used in no more than 2 studies were defined as rare tissues. We plotted the number of unique HK genes in each study as a function of its rare-tissue number shown in Fig 6. The results indicate that the more rare tissues are included in a study, the less unique HK genes are determined. This observation supports our notion that the incomplete sampling can increase the chance of identifying study-specific HK genes (likely false positives). Therefore this result encourages the merging of different studies to eliminate unauthentic ones.

To further examine the impact of studied tissues to HK genes, we defined Expression Breadth (EB). Different from DB (Detection Breadth), EB is to describe the sampling depth of HK-gene studies. Because the stringency of filtering criteria used in each study varied, we used EB and REB to quantify this variation as shown in Table 1.

### Functional enrichment Analysis

**Fig 7:**
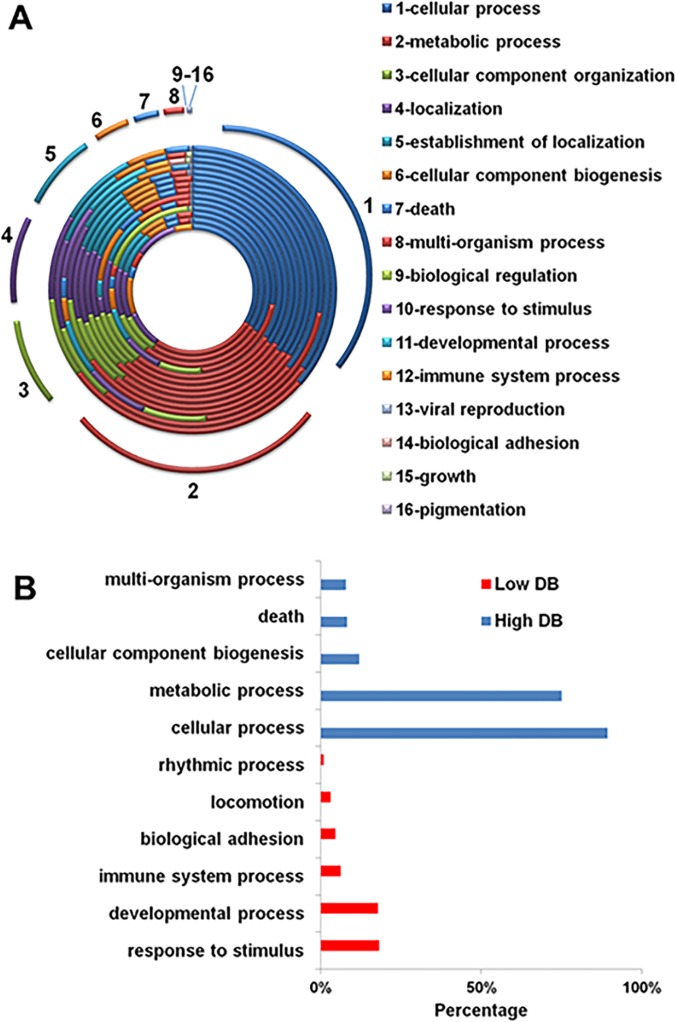
Gene ontology enrichment analysis of biological process of all the studies. From outer to inner circle, the displayed datasets are: “Fagerberg”, “Ramskold”, “Zhu_EST”, “Shyamsunder”, “Eisenberg_13”, “Reverter”, “Zhu_MA”, “Dezso”, “Chang”, “She”, “Tu”, “Podder”, “Warrington”, “Eisenberg_03”, and “Hsiao”.

Further our analysis, we examined the enriched functions of these genes. The results of the DAVID GO enrichment analysis are summarized in Fig 7A. In the figure, the percentage of genes in a particular GO_BP to the total GO genes in each study is plotted. Different from the results addressed above, BP enrichment displays a high consistency in all studies. In Fig 7A, almost all studies have the same enriched GO_BPs, and the proportion of these processes across studies is also similar.

To further examine DB impact to this enrichment, we separately analyzed the enriched BPs for high DB (≥10) and for unique genes (DB = 1), as shown in Fig 7B. The distribution of enriched functions in Fig 7B resembles that in Fig 7A, but the functions in high DB and DB = 1 groups are distinct. The unique-gene group enriches functions that are known to have large diversity, such as those related to immune response and cell surface adhesion; whereas the widely-detected genes carry more general and basal functions such as those related to metabolism, biogenesis and cell death.

## Discussion

Human HK-gene studies have been pursued by more than a dozen laboratories globally, and we for the first time try to integrate these studies with an aim to minimize the under-sampling bias. Our concern on under-sampling issue is raised from the HK gene definition.

The definition of HK gene is constantly evolving, and we have divided these definitions here into two major types as listed in Table 1. The early ones (Type I) represented by Watson et al. (1965) [24] and Warrington et al. (2000) [14] state that the HK genes need to be constitutively expressed in every tissue to maintain cellular functions. This definition has been widely used by several groups [8, 12, 15, 17, 25, 26]. Due to measurement errors and stochastic noise, it is difficult to distinguish genes absent in the sample from those weekly expressed, that are also called “expression leak”, a term used to describe the ubiquitous and trace expression of a large part of genome in all tissues[27]. A “cutoff level” has been proposed for HK genes[12], which requires relative quantification of all gene abundance in one sample and across all tissues studied. However, some HK genes such as transcription factors can have low expression, and a universal cutoff will prevent the identification of these genes. The newer definition (Type II) extends the Type I definition and emphasizes on a constant and stable expression instead of using a universal “cutoff”, which was initially raised by Butte et al.[1] and followed by Eisenberg and Levanon[3]. Type II definition allows lowly expressed genes to be included, and has gained popularity in recent studies[9, 10]. Furthermore, the sampling depth and the criteria used to determine constitutive expression have not been consistent in all the studies. We have used EB, REB, and number of rare tissues to quantify the stringency as summarized in Table 1. Based on both the Type I and Type II definitions and the actual tissues used, we reasoned that the more complete coverage of different tissues, the better elimination of potential false positive HK genes. Our correlation analysis on the rare tissue number and unique genes in Fig 6 confirmed this hypothesis.

In the process of integration, we translated all the identifiers to Entrez Gene ID. This step changed original datasets in some degree. Most datasets were expanded slightly as indicated in Table 1, because we had considered all the possible matching IDs during translation. This expansion should increase the chance of finding common genes across studies; however, the results were opposite.

Overall in Fig 1, an exponential decrease of common genes was observed with the increased number of the comparing datasets. Only one gene (peroxiredoxin 1, PRDX1) was found common in all, and 17 genes were shared by 14 of 15 studies (Fig 1, red bar). Even though we were expecting a smaller number in the merged list than hundreds to thousands of HK genes included in individual studies, yet we never expected it to be so small. The trend in Fig 1 suggests no convergence, meaning if more studies are introduced, common genes will drop to zero. On the contrary, genes shared by fewer studies grew exponentially suggesting difference is common in all studies.

To identify the cause of difference, we first performed the discrepancy analysis by examining the number of unique HK genes (Fig 2) followed by a relationship analysis using hierarchical clustering (Fig 3) and a deviation analysis using leave-out approach (Fig 1 and Table 1). We then performed pairwise similarity comparison (Fig 4). To examine the impact of detection sensitivity to the observed results, we further explored expression quantity (Fig 5). In the end, we concerned the enriched biological functions (Fig 7). Through the analyses conducted on common and unique genes, we discuss below the extrapolated information.

### Influence of HK Gene definition

The “Shyamsundar” list ranks the highest in leave-out deviation studies in Table 1, has the second highest number of unique genes in Fig 2, is the most distant study in hierarchical clustering in Fig 3, and has the second lowest overall similarity ratio in Fig 4. The observed large difference of this list is likely owing to the criteria Shyamsunder et al. used to derive the list. Because the study of Shyamsundar et al aimed to study common genes showing variable expression in different tissues but not necessarily HK genes, their filtering criterion agreed with Type I but against Type II definition. Specifically, they used a cutoff of at least four-fold variation to the mean expression and a REB of 75%, i.e. the lowest REB among all the studies included (Table 1).

In another case, “Fagerberg” shows the largest list in all studies, is the 2^nd^ most distant in hierarchical clustering, and contributes the most to the low-quantity genes in Fig 5A. Similar to “Shyamsundar”, the study of Fagerberg et al. was not focusing on HK genes but tissue specific expression. Even though all the genes in the “Fagerberg” list had been detected in all the tissues used in their study, Fagerberg et al. did not apply the Type II definition to stringently filter genes with potentially leakage expression for the consideration of HK genes.

In addition, Podder et al. also employed slightly different filtering criteria than others to derive their HK genes even though they had followed Type II definition. In their study, instead of applying separate filtering parameters for constitutive and stable expression, they used tissue specificity index τ [28] to consider the two factors simultaneously. In addition, Podder et al. used a relatively low cutoff, i.e. τ < 20%, to filtering HK genes. As a result, their study showed obvious deviation from the rest in various analyses. For example, “Podder” has the lowest overall similarity ratio in pairwise comparison, is the 3^rd^ distant study in hierarchical clustering (Fig 3), and ranks the 3^rd^ in leave-out deviation analysis (Table 1). The clear isolation of “Shyamsundar”, “Fagerberg”, and “Podder” from the rest in Fig 3 suggests the impact of definition to the HK-gene result and also demonstrates the sensitivity and reliability of our analyses.

### Complexity in the cause of the discrepancy

After identifying the definition influence, we had anticipated that the removal of these highly deviated studies can drastically increase the common-gene number. However, the removal of the top two most deviated lists (“Shyamsundar” and “Podder”) in leave-out analysis (Fig 1) only slightly increased the common genes from 1 to 20. The gain of common genes in leave-out analysis surpassed that in DB analysis, only when the majority of studies (12 out of 15 studies) were dropped. This result indicates that the cause to the observed discrepancy spreads across all studies. In another words, the divergence among datasets is complex and is contributed by more factors than the definition itself.

This notion has been further confirmed in the pairwise similarity analysis (Fig 4). In Fig 4, the range of similarity ratio obtained from any pair in general was modestly high with a mean of 0.62, which agrees with literature reports of close to 60% similarity when comparing a few studies[2, 6, 8, 10, 22]. This agreement further demonstrates the reliability of our analysis. In Fig 4, all the top three largest lists (“Fagerberg”, “Ramskold”, and “Zhu_EST”) used sequencing based detection, and have the high overall similarity ratios as indicated by the relatively red bands, suggesting the influence of the list size and the technique to the comparison. Similarly, the studies with relatively small number of total HK genes and used the same microarray chips also form a red island in Fig 4, represented by “Warrington” and “Hsiao”, “Chang”, “Tu” and “Zhu_MA”. For the variations shown within the red and blue regions in Fig 4, many other factors can also contribute such as the studied tissue types. To break down the observed complexity, we further examined factors such as gene abundance, detection techniques, analyzed tissue types, and the enriched functions.

### Influence of gene abundance

Gene expression level, i.e. gene abundance, can influence the likelihood that a gene is detected. We observed more than three orders of magnitude of dynamic difference in mean gene abundance from 8 lists having quantitative information. Fig 5A suggests a large number of HK genes are low in abundance, yet most of these genes are from the “Fagerberg” list. Because of the study purpose difference as discussed above, we conducted another similar analysis with “Fagerberg” list removed, and the result is shown in S1 Fig panel A. A similar pattern but fewer low-quantity genes (< 0.2) than those in Fig 5A was observed, suggesting low-expression HK genes do exist but may not be as many as shown in Fig 5A.

In Fig 5B, the analysis of gene abundance to DB shows that both high and low DB genes are abundant. In S1 Fig panel B after the removal of “Fagerberg”, a similar pattern with more obvious increase of gene expression at low DB is observed. The observation of relatively high expression for low DB genes indicates that the detection limit of the used technical platforms is unlikely the reason for most studies to miss low DB genes. Nevertheless, both hierarchical clustering and pairwise comparison showed that results from similar techniques were closer to one another. Therefore, we decided to further investigate the impact of detection methods to the obtained results.

### Influence of experimental methods

We summarized the techniques used in each study in Table 1 and S1 Text. These techniques include Microarray (MA)[2, 8–11], RNA-sequencing (RNA-seq)[3, 12], Expressed Sequence Tag (EST)[6] and Massive Parallel Signature Sequence (MPSS)[29]. Due to the difference in working principles, biases of these techniques have been discussed in various publications[30, 31]. For instance, MPSS and EST are both based on sequencing of the amplified tags located in the specific transcriptional loci (usually 3' or 5' ends)[32, 33]. Transcripts are confirmed only when they match the known loci, thereby excluding novel genes[34–37]. In addition, EST is sensitive to cloning biases and has relatively low throughput [36, 38, 39]. Microarray technique, on the contrary, is a hybridization-based platform. The method heavily relies on the prior knowledge of genes to design probes, a step which is prone of biases especially for early microarrays[4, 37]. Tiling array is possible to discover novel transcripts[40, 41], yet the high cost of covering the entire genome limits its use in HK-gene discovery. The hybridization efficiency, biases, and the limited dynamic range have plagued microarray technology for highly sensitive and quantitative analyses[31, 42].

Among all the existing trancriptomic techniques, RNA-seq has the highest throughput, dynamic range, and the most accuracy. These merits allow RNA-seq to minimize many of the drawbacks addressed above [42–45]. RNA-seq is also a sequencing based technique that reads cDNA fragments reversely translated from the sample transcriptome, therefore does not require the prior knowledge of a gene. The high sensitivity of this technique allows the identification of expression leak[38, 42] that is common to all tissues and organs. The advent of this technique has in part prompted the transformation of the HK gene definition from Type I to Type II[1–3, 23, 46] as we discussed above. However, the short reads in RNA-seq affect accurate gene alignment[47–49] especially for HK genes, among which short repeats are more frequent than non-HK genes[50, 51]. This difficulty also impairs quantification accuracy[52–54], even though the dynamic range of RNA-seq can reach five orders of magnitude[42].

We observed high degree of consistency between detection technique and clustering in Fig 3. The absence of “Dezso”, “She” and “Eisengerg_03” in the microarray subclusters of 2.1 and 2.2 can be explained by their use of different chips than the ones in the subclusters. “Dezso” used ABI Human Genome Survey array[8], “She” used a customized chip[9], and “Eisenberg_03”[2] used the early microarray results of Su et al. [55]. Also considering the information learned in Fig 5B that detection limit should not be the factor preventing the identification of DB = 1 genes, the observed differences between microarray and other sequencing based technique as well as the differences within microarray platform is likely contributed by probe bias.

We also recognized that all techniques have experienced fast development in recent years. For microarray alone, the number of probes on a chip has increased about one fold, from only ~ 12000 gene probes to 22,000 gene probes[56–59]. As a result, the number of HK genes also increased in latest studies (Table 1). For RNA-seq that had suffered from short reads and alignment challenges [12], its resent capability to read long 50–100 bases [3,17] have minimized the error rate. Some of these errors and technical biases are likely contributed to the observed variations.

Comparing to HK-gene definition, technical bias seem to outperform definition difference for certain results. For example, in Fig 3 hierarchical clustering, the largest distance is contributed by the definition difference, yet in both two main clusters, i.e. sequencing cluster 1 and microarray cluster 2, a mixture of Type I and II definitions exists.

### Influence of biological variation

Besides the definition and methodology differences, we examined the under-sampling concern raised at the beginning of the study. We wanted to know what types of samples each study used, and how the sample difference affected the observed discrepancy. Even though the definition of HK genes implies the robust expression with little environmental and biological impact, it is known that different tissue types exhibit drastic anatomical and physiological differences. At the same time, tissues at different developmental and disease stages experience profound changes in gene profiles and protein networks [16]. Therefore incomplete tissue sampling can result in inflated HK genes. Because in many studies concerned here, such as “Warrington”, “Eisenberg_03”, “Eisenberg_13”, “Tu”, and “She”, their tissue types include not only normal adult tissues, but also fetal or cancer tissues (S2 Table), we asked whether the biological difference in analyzed samples can contribute to the observed discrepancy.

Table 1 summarizes the number of rare tissues used in each study. S2 Table tabulates the details. Together 187 distinct types have been studied accounting for more than 90% of estimated tissue and cell types. We examined the correlation between the used rare tissues and the obtained unique genes as shown in Fig 6. In the figure, the unique gene number is reversely proportional to the rare tissue number, i.e. the more rare tissues used, the less unique genes identified. In theory, the deeper the sampling depth will have the less false positive HK genes owing to more complete sample coverage. The observed drop of unique genes in more comprehensive studies is, therefore expected and confirms the existence of inflation in all HK-gene studies. The slight deviation of “Shyamsundar” from the rest further emphasizes the sensitivity of this analysis.

Collectively, these results suggest that complex factors including the HK-gene definition, filtering criteria, detection and sampling have contributed to the observed small overlap of all studies. We then further examined the biological functions of HK genes.

### Housekeeping Functions

As the definition of HK genes regardless Type I or II, emphasizes on basal functions supported by these genes, we deliberately examined the enriched GO_BPs as shown in Fig 7. Irrespective to the scarce common genes in all datasets, we observed very conservative functions across studies. The enriched GO_BPs included cellular and metabolic processes, cellular component organization and biogenesis among other key basal functions such as cell death that are consistent with previous reports [12, 60–63].

We are surprised to observe these consistent functions from such diverse lists. To further verify our observations, we analyzed a well-known and key biological process, i.e. glycolysis, in all the derived HK-gene lists. Glycolytic enzymes have been known to carry less variations than other random genes[64]. It is interesting to use this conserved pathway to probe the observed diversity in all datasets. We performed a pairwise comparison to obtain the identification rate of this pathway in all HK lists as shown in Fig 4. The overall mean identification rate is 32±18%. Among all the lists, “Zhu_EST” has the highest identification rate of 66%, whereas “Podder” has the lowest value of 5%. The most common gene is “glucose phosphate isomerase” shared by 9 out of 15 lists. Even for such a conversed function, the variation can be clearly observed among different lists, which validated our conclusion, i.e. the HK genes obtained so far do not converge.

Given the fact that HK functions are much more consistent than the actual HK genes in all studies, and that the inflation of HK genes seems ubiquitous in all datasets, we start to question whether it is possible that the bona fide HK genes do not exist, or exist in a much smaller number than what have been reported (i.e. hundreds and thousands). We believe that the majority of the HK genes discovered so far can be conditional, i.e. expressed only in a subset of tissues at specific biological stage under certain environment, instead of constitutive and ubiquitous.

Several pieces of evidence support our belief. First, even though HK genes are difficult to verify, especially human HK genes, experimentally and computationally the minimum number of essential genes has been widely studied[65–69]. The essentiality of a gene can be obtained through knockout experiments on animal embryos or embryonic stem cells[70–72]. Because of the similarity in definition of the essential genes and HK genes, the knowledge on essential genes will shine light on HK genes. [5, 73]. Experiments conducted on essential genes indicated that most essential genes in early development showed later tissue specificity instead of housekeeping. The tissues absent of the essential genes usually express alternatives carrying similar basal functions. These observations support our notion that it may be more appropriate to ascribe HK functions instead of HK genes for maintenance purpose in all tissues at all stages.

Secondly, our belief is supported by the view taken from the evolution perspective. In the process of organ and tissue specification, gene identity and function have been largely diversified, a result that is manifested by the numerous yet discrete morphologies and behaviors of different cell types that observed in anatomy and physiology. These distinct cell types, are connected by complex and cohesive interactions that give rise to new functions, to allow individual multicellular organism gain robustness and flexibility (adaptation) that can be achieved by a population of single-cellular organisms [74, 75]. One of the key factors in evolution is the diversification of genes and gene products. The large human transcriptome and proteome derived from a relatively small population of about 20,000 genes enable the execution of same function through different gene products, an event that is frequently seen at all levels of biological hierarchy.

The utilization of different gene products for the same function is not only molecularly sufficient, but also necessary. It is advantageous in survival and adaptation for organisms to use slightly different sets of genes or gene products in different tissues. This diversity can effectively avoid catastrophic and fatal events targeting to common genes shared by all the cells at all developmental stages. As a result, we believe that the absence of HK genes is evolutionarily favored for multicellular organisms to survive and thrive.

Our observation also raises the importance to study gene products at both transcript and protein level. In the past, for simplicity, studies tend to converge different transcripts and proteins to genes [76]. Yet it has been clear that transcription is a complex process, in which extensive overlap exists in transcriptional units, and alternative splicing has largely increased the transcript repertoire. At protein level, rich translational and post-translational modifications have further extended the diversity of gene products, and many these modifications are key to functions. Same gene yet different splicing forms or different post-translational modifications can carry different even opposite functions[77]. Therefore, it is important to address the exact sequence and structure information of gene products instead of simply using genes for easy of study.

Thirdly, the likelihood of no HK genes has been further indicated in our understanding of control genes, which is another branch of HK-gene studies. Due to the need in quantitative gene analysis, stably expressed control genes are necessary to normalize different biological samples used for comparison. HK genes have been widely deployed for these purposes. Nevertheless, recent accurate analyses consistently showed that these control genes had a large range of expression variation and were sensitive to study conditions [60, 78–80]; therefore it was recommended that for each study, the choice of control genes needed to be experimentally verified for their stable expression [81–84]. The observation on control genes further implies that it will be extremely challenging to find any gene with stringent stable and constant expression.

In summary, from our functional analysis, from the experimental information gained in essential genes, from the knowledge of control genes in quantitative analysis, and from the condition for best survival, the bona fide HK genes based on current definition might not exist. We recommend HK genes to be defined under well described conditions such as cell types, growth stages, cell cycles as well as various physiological and environmental conditions with consideration of specific splice variants and protein modifications; or we recommend of using more consistent HK functions instead. This notion does not defy the effort to quest HK genes. In fact, regardless of the actual gene-identity variation, all the HK-gene studies have universally identified many interesting characteristics shared by HK genes. These characteristics range from the slow evolution rate[25, 85], the compact structure[2, 20], to unique transcriptional and translational regulations[86–89]. Without the existing high quality studies, our work here would not be possible. The knowledge acquired from HK gene studies on genomic structure and function has greatly benefited our understanding of health and diseases. With the increased knowledge of gene variation in biological system, a shift to HK function from HK genes may provide freedom for easier accumulation of more interesting findings.

At last, regardless the existence of HK genes, our study here resolved a list of genes with relatively broad tissue expression (DB> = 13) as shown in Table 3. These genes show high similarity to the published loading control gene lists[3, 46, 90–95], and are mostly concentrated on the ribosome, mitochondria, and proteasome genes. We hope this list can enrich the current control gene pool for various quantitative biological studies.

**Table 3:** Control gene candidates with DB ≥ 13*.

Gene ID	Symbol	Locus	Accession	Orientation	Exon count	OMIM
**5052**	PRDX1	1p34.1	NC_000001.11	minus	7	176763
**7316**	UBC	12q24.3	NC_000012.12	minus	2	191340
**7314**	UBB	17p12-p11.2	NC_000017.11	plus	5	191339
**1936**	EEF1D	8q24.3	NC_000008.11	minus	15	130592
**292**	SLC25A5	Xq24	NC_000023.11	plus	4	300150
**6118**	RPA2	1p35	NC_000001.11	minus	9	179836
**6132**	RPL8	8q24.3	NC_000008.11	minus	7	604177
**6135**	RPL11	1p36.1-p35	NC_000001.11	plus	6	604175
**6141**	RPL18	19q13	NC_000019.10	minus	7	604179
**6169**	RPL38	17q25.1	NC_000017.11	plus	5	604182
**6185**	RPN2	20q12-q13.1	NC_000020.11	plus	19	180490
**6193**	RPS5	19q13.4	NC_000019.10	plus	6	603630
**6194**	RPS6	9p21	NC_000009.12	minus	6	180460
**6203**	RPS9	19q13.4	NC_000019.10	plus	7	603631
**6217**	RPS16	19q13.1	NC_000019.10	minus	4	603675
**6223**	RPS19	19q13.2	NC_000019.10	plus	6	603474
**6229**	RPS24	10q22	NC_000010.11	plus	10	602412
**6118**	RPA2	1p35	NC_000001.11	minus	9	179836
**334**	APLP2	11q24	NC_000011.10	plus	19	104776
**375**	ARF1	1q42	NC_000001.11	plus	6	103180
**498**	ATP5A1	18q21	NC_000018.10	minus	13	164360
**518**	ATP5G3	2q31.1	NC_000002.12	minus	4	602736
**567**	B2M	15q21.1	NC_000015.10	plus	4	109700
**801**	CALM1	14q32.11	NC_000014.9	plus	7	114180
**805**	CALM2	2p21	NC_000002.12	minus	6	114182
**808**	CALM3	19q13.2-q13.3	NC_000019.10	plus	6	114183
**967**	CD63	12q12-q13	NC_000012.12	minus	12	155740
**5573**	PRKAR1A	17q24.2	NC_000017.11	plus	14	188830
**5692**	PSMB4	1q21	NC_000001.11	plus	7	602177
**5693**	PSMB5	14q11.2	NC_000014.9	minus	5	600306
**5714**	PSMD8	19q13.2	NC_000019.10	plus	7	
**1176**	AP3S1	5q22	NC_000005.10	plus	8	601507
**1340**	COX6B1	19q13.1	NC_000019.10	plus	4	124089
**1347**	COX7A2	6q12	NC_000006.12	minus	4	123996
**1350**	COX7C	5q14	NC_000005.10	plus	3	603774
**1476**	CSTB	21q22.3	NC_000021.9	minus	3	601145
**1603**	DAD1	14q11.2	NC_000014.9	minus	3	600243
**1655**	DDX5	17q21	NC_000017.11	minus	15	180630
**1938**	EEF2	19p13.3	NC_000019.10	minus	15	130610
**1982**	EIF4G2	11p15	NC_000011.10	minus	23	602325
**2079**	ERH	14q24.1	NC_000014.9	minus	4	601191
**2665**	GDI2	10p15	NC_000010.11	minus	11	600767
**2778**	GNAS	20q13.3	NC_000020.11	plus	17	139320
**3020**	H3F3A	1q42.12	NC_000001.11	plus	4	601128
**3021**	H3F3B	17q25.1	NC_000017.11	minus	4	601058
**3094**	HINT1	5q31.2	NC_000005.10	minus	5	601314
**3146**	HMGB1	13q12	NC_000013.11	minus	8	163905
**3735**	KARS	16q23.1	NC_000016.10	minus	15	601421
**3939**	LDHA	11p15.4	NC_000011.10	plus	9	150000
**4673**	NAP1L1	12q21.2	NC_000012.12	minus	16	164060
**4691**	NCL	2q37.1	NC_000002.12	minus	14	164035
**4738**	NEDD8	14q12	NC_000014.9	minus	4	603171
**975**	CD81	11p15.5	NC_000011.10	plus	9	186845
**5094**	PCBP2	12q13.13	NC_000012.12	plus	15	601210
**5230**	PGK1	Xq13.3	NC_000023.11	plus	11	311800
**5441**	POLR2L	11p15	NC_000011.10	minus	2	601189
**5501**	PPP1CC	12q24.1-q24.2	NC_000012.12	minus	10	176914
**6647**	SOD1	21q22.11	NC_000021.9	plus	5	147450
**6651**	SON	21q22.11	NC_000021.9	plus	16	182465
**6727**	SRP14	15q22	NC_000015.10	minus	6	600708
**6746**	SSR2	1q21-q23	NC_000001.11	minus	6	600867
**8892**	EIF2B2	14q24.3	NC_000014.9	plus	8	606454
**9168**	TMSB10	2p11.2	NC_000002.12	plus	3	188399
**9296**	ATP6V1F	7q32	NC_000007.14	plus	3	607160
**9802**	DAZAP2	12q12	NC_000012.12	plus	5	607431
**10109**	ARPC2	2q36.1	NC_000002.12	plus	11	604224
**10399**	GNB2L1	5q35.3	NC_000005.10	minus	8	176981
**11315**	PARK7	1p36.23	NC_000001.11	plus	8	602533

* pseudo genes are removed.

In the end, we hope our observation and explanation can bring some new perspective in examining HK genes. We want to emphasize the importance and necessity of existing studies, and their relentless release of all their data to make the current analysis possible. We also want to emphasize the usefulness of revisiting published data for novel insight, which in our opinion helps to maximize the value of the past work.

## Supporting Information

S1 Fig (A) Distribution of HK-gene population as a function of normalized gene-expression quantity after removing “Fagerberg”. (B) Distribution of the detection breadth (DB) as a function the normalized gene-expression quantity. Error bar represents the standard deviation after removing “Fagerberg”.
(TIF)

S1 Table Websites of the downloaded datasets. (XLSX)

S2 Table Tissue and cell types used in each study. (XLSX)

S1 Text Summary of experimental conditions and filter criteria of each study. (PDF)
