# Supplementary figures and images for: Do Housekeeping Genes Exist?
Source: PLoS One. 2015 May 13;10(5):e0123691. doi: 10.1371/journal.pone.0123691 (PMC4430495; doi:10.1371/journal.pone.0123691)

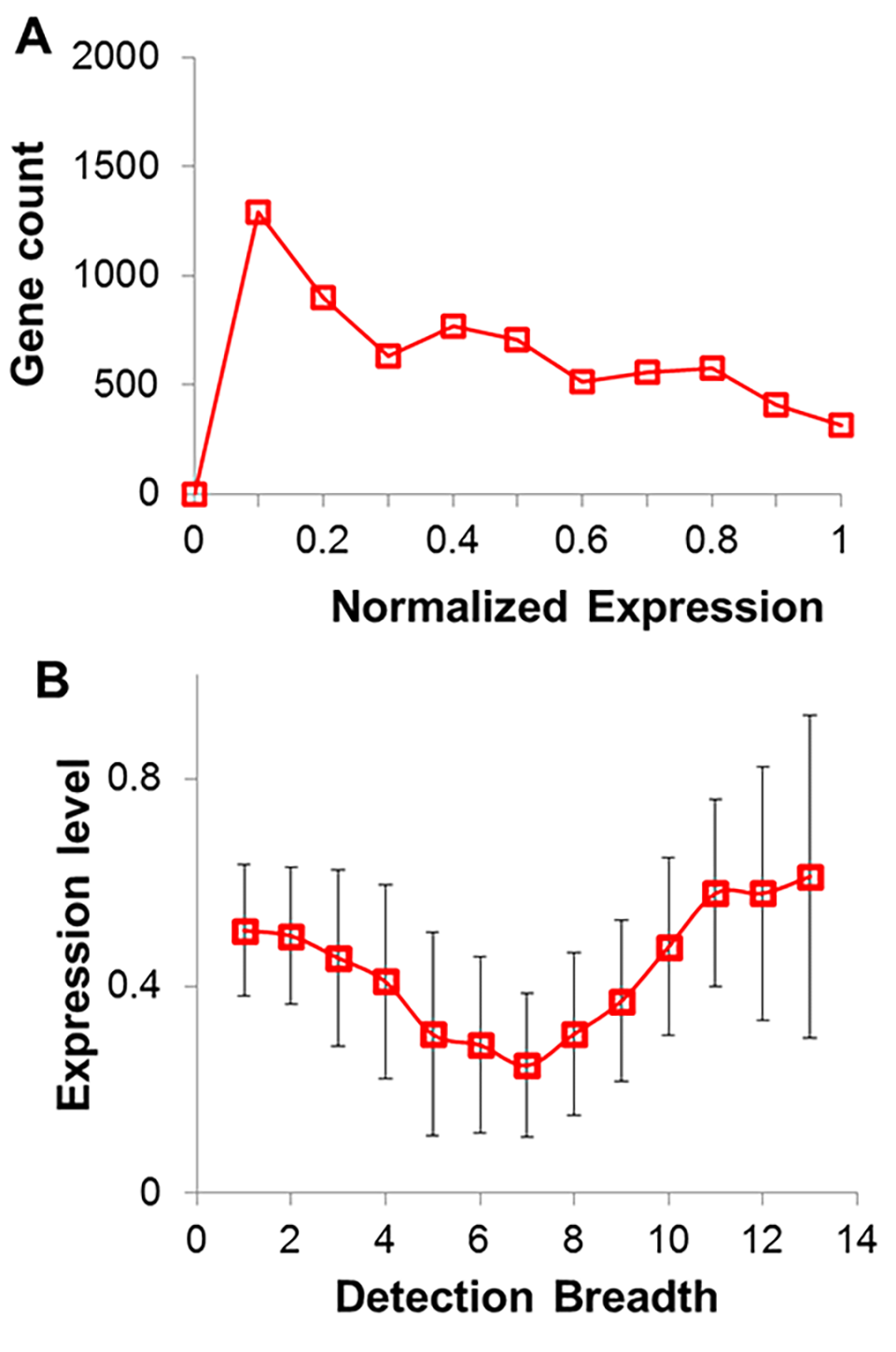

Supplement: S1 Fig — (B) Distribution of the detection breadth (DB) as a function the normalized gene-expression quantity. Error bar represents the standard deviation after removing “Fagerberg”. (TIF) [file pone.0123691.s001.tif]
