# Supplementary material for: Do Housekeeping Genes Exist?
Source: PLoS One. 2015 May 13;10(5):e0123691. doi: 10.1371/journal.pone.0123691 (PMC4430495; doi:10.1371/journal.pone.0123691)
Supplement: S1 Text — (PDF) [file pone.0123691.s004.pdf]

Do Housekeeping Genes Exist?

Yijuan Zhang, Ding Li, and Bingyun Sun

Supplementary text:

"Warrington": the study used HuGeneFL GeneChip probe microarray (7,129 genes and 25 mer oligonucleotide probes) and characterized 60 human adult and fetal samples respectively for 11 different tissue types. All samples were prepared and hybridized in duplicate. Analysis was done using GeneChip 3.0, and only transcripts detected as present in duplicate hybridizations or absent in duplicate hybridizations were reported. Genes expressed in all tissues were defined as HK genes with expression breadth (EB) of 11, and relative expression breadth (REB) of 100%.

"Hsiao": the study used HuGeneFL GeneChip microarray (7,070 unique sequences) and characterized 59 human samples of 19 different tissue types, from 49 individuals including 24 males and 25 females with normal histology. Analysis was done using GeneChip 3.1, and genes with a present call in at least one sample of each tissue type were included.   Again, genes expressed in all tissues were defined as HK genes with expression breadth (EB) of 19, and relative expression breadth (REB) of 100%.

"Eisenberg_03": the study used the results of Su et al [1], in which Affymetrix U95A high-density oligonucleotide microarray (12,600 probes) was used to study 101 different samples from 47 human tissues and cell lines that are mostly normal. The Eisenberg et al. unified all probes measuring the same gene and ignored the potential

splice variants for a final of 7,500 human genes. The study used GeneChip 3.2 software and a standard Affymetrix average difference unit of 200 as cutoff for the present call. Same as above, genes expressed in all tissues were defined as HK genes with expression breadth (EB) of 47, and relative expression breadth (REB) of 100%.

"Tu": the study used another result of Su et al [2], in which a combination of U133Aand GNF1H microarrays (33,698 human genes) were used to interrogate 160 samples from 79 human tissues. The cutoff of a present call in this study was 550 standard Affymetrix average difference units, and HK genes were filtered with expression in at least 73 of 79 tissues with expression breadth (EB) of 79, and relative expression breadth (REB) of 92%.

"Zhu": the study also used the microarray results of Su et al [2] published in 2004. In addition, "Zhu" results also included 8 million human ESTs complied from 4,026 RNA (or tissue and organ) samples downloaded from UCSC annotation database [3]. To compare, Zhu et al. chose 18 well-studied tissues covered by both data types (i.e. microarray and EST). For EST data, the call of presence is defined as expressed in a given tissue when at least one reliable EST or an EST cluster was detected from that tissue. For microarray data, the expression was defined by a cutoff value of 200. To maximize the overlapping potential, we used their upper bound of filtering HK genes, i.e. genes expressed in 16 out of 18 tissues with expression breadth (EB) of 18, and relative expression breadth (REB) of 89%.

“Podder”: the study used 41 human EST libraries retrieved from TIGR Gene Indices (http:// compbio.dfci.harvard.edu/tgi/) after removing 138 pathogenic and cancerous libraries. Sequence match was carried out by obtaining at least 60% identity and 80% overlaps to human-specific ESTs, and the sum of EST count across all 41 libraries was used to represent the mRNA abundance.

For filtering HK genes, this study used tissue specificity index τ [4]. The τ of a human gene i is defined by

$$\tau_H = \frac{\sum_{j=1}^{n_H}(1 - [\frac{\log_2 S_H(i,j)}{\log_2 S_H(i,\max)}])}{n_H - 1}$$

where n_H is the number of human tissues examined and S_H (i, max) is the highest expression signal of gene i across the n_H tissues. The value ranges from zero to one, with higher values indicating higher variations in expression level across tissues or higher tissue specificities. If a gene has expression in only one tissue, the value approaches to one. In contrast, if a gene is equally expressed in all tissues, τ =0. This study assigned housekeeping to genes with τ having the lowest 20% of population.

“Dezso”: the study used Applied Biosystems Human Genome Survey Microarray (P/N 4337467) contained 31,700 60-mer oligonucleotide probes. A total of 31 different normal human tissues were characterized and each with 3 technical replicates. Applied Biosystems 1700 Chemiluminescent Microarray Analyzer software v1.1 (P/N 4336391) was used for data analysis. The cutoff of present call was determined on a probe-by-probe basis across all three technical replicates. Genes consistently expressed

in all tissues were filtered for the housekeeping set with expression breadth (EB) of 31, and relative expression breadth (REB) of 100%.

"She": the study used customized high density microarray (18,149 genes) and characterized gene expression profiles of 42 normal human tissues that were pooled from multiple donors (typically twelve). Most tissues were from adults, yet four fetal tissues of brain, kidney, liver and lung were also included. The filtering criteria of "She" results were: the intensity of the gene must be greater than the overall median intensity of all genes in the microarray in at least 41 out of 42 tissues and the coefficient of variance of the gene intensity across tissues must be less than 1. We considered the EB of 42 and REB of 98% (41/42*100%) for this study.

"Chang": the study compiled 1,431 raw files of Affymetrix GeneChip microarray HGU133A or HG-U133-Plus2 (22,277 common probe sets mapped to 12,559 unique Entrez Gene IDs) from 104 publicly available data sets on 43 normal human tissues[5]. Each tissue type had at least 5 samples. The mean intensity of each probe set was scaled to 200 for comparison. To filter HK genes, this study weighted expression intensity with fraction Present for each gene as following:

$$FPEI_{ij} = EI_{ij} \times FP_{ij}$$

where $FPEI_{ij}$ is the fraction Present weighted expression intensity (FPEI) for gene i in tissue type j, which combines the expression abundance EI_{ij} and the confidence of detection FP_{ij}. Genes of $FPEI_{ij} > 100$ in all 43 studied tissues were defined as HK genes,

therefore the EB of this study is 43 and REB is 100%.

"Shyamsundar": the study used a customized microarray (26,260 genes) and characterized 115 normal human samples of 35 different tissue types. Fluorescence ratios from the microarray were extracted using GenePix software. This study concerned well-measured genes whose expression varied, as determined by: signal intensity over background more than twofold in either test or reference channels in at least 75% of samples; and a fourfold or more ratio variation from the mean in at least two samples. We used variably expressed genes for HK gene analysis with EB of 35 and REB of 75%.

"Ramskold": the study used short read RNA-seq results from Wang et al. (SRA002355.1)[6], Marioni et al. [7] and Mudge et al.[8]. The human samples concerned include 18 different types of tissues and cell lines at a depth of roughly 20 million short reads per sample. The gene expression cutoff is 0.3 reads per kilobase of exon model per million mapped reads (RPKM). Genes expressed in all tissues were defined as HK genes with expression breadth (EB) of 18, and relative expression breadth (REB) of 100%.

"Reverter": the study used data from massively parallel signature sequencing (MPSS) results collected by Jongeneel et al. [9] with 182,719 tag signatures across 32 tissues. The present call is for tags expressed at more than 5 transcripts per million (tpm) in all

tissues. HK genes were filtered by expression in more than 25 out of 32 tissues, i.e. EB of 32 and REB of 78%.

“Eisenberg_13”: the study analyzed RNA-seq expression data from the Human BodyMap (HBM) 2.0 Project and characterized 16 normal human tissue types. Sequencing was performed on HiSeq 2000 instruments, and two different read lengths were used for each tissue (2×50-bp paired-end and 1×75-bp single-read data). Bowtie2 [10] was used for sequence alignment, and quantitation was based on normalized RPKM units. Filtering criteria for HK exons were 1) expression observed in all tissues; 2) low variance over tissues: standard-deviation [$\log_2$(RPKM)]<1; and (3) no exceptional expression in any single tissue; that is, no log-expression value differed from the averaged $\log_2$(RPKM) by two or more. The HK genes were filtered for at least half of its exons being housekeeping exons with EB of 16 and REB of 100%.

“Fagerberg”: the study used RNA-seq and characterized the transcriptomes of 27 different normal human organs and tissues. Sequencing was performed on HiSeq2000 and HiSeq2500 machines with a read length of 2×100 bases. Raw reads below a phred quality of 20 were trimmed using the software sickle[11], and reads shorter than 54mers were discarded. Tophat v.2.0.3 was used for sequence alignment, and the quantitation was based on the fragments per kilobase of exon model per million mapped reads (FPKM) using Cufflinks v2.0.2 [12]. A cutoff value of 1 FPKM was used as the present call. HK genes were filtered by constitutional expression in all the tissue and organ

types, with EB of 27, and REB of 100%.

References

1. Su AI, Cooke MP, Ching KA, Hakak Y, Walker JR, et al. (2002) Large-scale analysis of the human and mouse transcriptomes. Proceedings of the National Academy of Sciences 99: 4465-4470.

2. Su AI, Wiltshire T, Batalov S, Lapp H, Ching KA, et al. (2004) A gene atlas of the mouse and human protein-encoding transcriptomes. Proceedings of the National Academy of Sciences of the United States of America 101: 6062-6067.

3. Kuhn RM, Karolchik D, Zweig AS, Trumbower H, Thomas DJ, et al. (2007) The UCSC genome browser database: update 2007. Nucleic acids research 35: D668-D673.

4. Yanai I, Benjamin H, Shmoish M, Chalifa-Caspi V, Shklar M, et al. (2005) Genome-wide midrange transcription profiles reveal expression level relationships in human tissue specification. Bioinformatics 21: 650-659.

5. Cheng W-C, Tsai M-L, Chang C-W, Huang C-L, Chen C-R, et al. (2010) Microarray meta-analysis database (M2DB): a uniformly pre-processed, quality controlled, and manually curated human clinical microarray database. BMC bioinformatics 11: 421.

6. Wang ET, Sandberg R, Luo S, Khrebtukova I, Zhang L, et al. (2008) Alternative isoform regulation in human tissue transcriptomes. Nature 456: 470-476.

7. Marioni JC, Mason CE, Mane SM, Stephens M, Gilad Y (2008) RNA-seq: an assessment of technical reproducibility and comparison with gene expression

arrays. Genome research 18: 1509-1517.

8. Mudge J, Miller NA, Khrebtukova I, Lindquist IE, May GD, et al. (2008) Genomic convergence analysis of schizophrenia: mRNA sequencing reveals altered synaptic vesicular transport in post-mortem cerebellum. PloS one 3: e3625.

9. Jongeneel CV, Delorenzi M, Iseli C, Zhou D, Haudenschild CD, et al. (2005) An atlas of human gene expression from massively parallel signature sequencing (MPSS). Genome research 15: 1007-1014.

10. Langmead B, Salzberg SL (2012) Fast gapped-read alignment with Bowtie 2. Nature methods 9: 357-359.

11. quality hgcnsS-AwattfFfu.

12. Trapnell C, Williams BA, Pertea G, Mortazavi A, Kwan G, et al. (2010) Transcript assembly and quantification by RNA-Seq reveals unannotated transcripts and isoform switching during cell differentiation. Nature biotechnology 28: 511-515.
